# Single Multiplex Polymerase Chain Reaction To Detect Diverse Loci Associated with Diarrheagenic *Escherichia coli*

**DOI:** 10.3201/eid0901.01-0507

**Published:** 2003-01

**Authors:** Catalina López-Saucedo, Jorge F. Cerna, Nicolas Villegas-Sepulveda, Rocío Thompson, F. Raul Velazquez, Javier Torres, Phillip I. Tarr, Teresa Estrada-García

**Affiliations:** * Centro de Investigación y de Estudios Avanzados del Instituto Politécnico Nacional, México D.F., México; † Hospital de Pediatría, México D.F., México; ‡ Children’s Hospital and Regional Medical Center, Seattle, Washington, USA; § University of Washington School of Medicine, Seattle, Washington, USA

**Keywords:** Diarrheagenic Escherichia coli, multiplex PCR, food safety, diagnostic microbiology, dispatch

## Abstract

We developed and tested a single multiplex polymerase chain reaction (PCR) that detects enterotoxigenic, enteropathogenic, enteroinvasive, and Shiga-toxin–producing *Escherichia coli*. This PCR is specific, sensitive, and rapid in detecting target isolates in stool and food. Because of its simplicity, economy, and efficiency, this protocol warrants further evaluation in large, prospective studies of polymicrobial substances.

*Escherichia coli* causes disease in humans through diverse mechanisms (*1*). Classified on basis of their virulence traits, the most well-studied members of the diarrheagenic *E. coli* group include enterotoxigenic *E. coli* (ETEC), enteropathogenic *E. coli* (EPEC), enteroinvasive *E. coli* (EIEC), enteroaggregative *E. coli* (EAggEC), and Shiga-toxin–producing *E. coli* (STEC), also called verocytotoxin-producing or enterohemorrhagic *E. coli*. ETEC produce secretory toxins (enterotoxins); EPEC adhere intimately to epithelial cells and induce host cell transmembrane signaling; EIEC invade eukaryotic cells; and STEC produce Shiga toxins.

Identifying diarrheagenic *E. coli* in the polymicrobial milieus of stool and food poses challenges. Occasionally, economically detectable phenotypes distinguish such organisms when they are abundant in human stools. For example, sorbitol- and lactose-nonfermenting colonies are typical of *E. coli* O157:H7 and EIEC (*2*,*3*), respectively. However, these phenotypes are nonspecific, and subsidiary testing is needed to confirm the isolate identity. In vitro assays that detect toxins, adherence, or invasion phenotypes can also identify candidate diarrheagenic *E. coli*. These determinations are often expensive, require special expertise, and employ various detection systems (e.g., cell culture, cytotoxicity assays). Applying such assays to enteric microbiologic diagnosis is cumbersome.

Nucleic acid hybridization techniques, exploited by colony hybridizations or polymerase chain reaction (PCR), apply a single detection method to a diversity of organisms. The application of nucleic acid amplifications requires selecting appropriate oligonucleotide primers and optimizing conditions to maximize sensitivity and specificity. The inclusion of reactions and conditions that apply to a variety of virulence loci so that multiple candidate pathogens can be sought in a single reaction makes this technology more efficient and economical. Such multiplex detection is an appropriate solution to the challenge of finding diarrheagenic *E. coli* in stools and in food. We describe the development of a multiplex PCR that detects four categories of diarrheagenic *E. coli* and the application of the assay to human diarrheal stools and food in Mexico City.

## The Study

We developed a single multiplex PCR reaction to detect ETEC, EPEC, EIEC, and STEC, using specific previously described (*4*–*6*) or new primers (GIBCO-BRL, Gaithersburg, MD) for diverse virulence traits (Table 1). Because primers for loci that unambiguously distinguish pathogenic from nonpathogenic EAggEC have not yet been determined (*1*), we did not address this group in this study.

**Table 1:** Prototypes and reference strains of ETEC, EPEC, EIEC, and STEC tested in multiplex PCR by using specific oligonucleotide primers for several locus^a^

*E. coli* category tested strains and serotypes	Locus	Primers	Amplicon size (bp)	Primer (pMol) in mix
**ETEC** H10407 O78:H11^b^ (*7*) E9034A O8:H9 (8) B_2_C O6:H16 (8) E8775A O25:H42^c^ (*9*)	*lt*	F:5´GGC GAC AGA TTA TAC CGT GC3´ (*4*) R:5´ CGG TCT CTA TAT TCC CTG TT3´ (*4*)	440	5.0
**ETEC** H10407 O78:H11^b^ (*7*) E9034A O8:H9 (*8*) B_2_C O6:H16 (*8*) E8775A O25:H42^c^ (*9*)	*st*	F:5´ATT TTT CTT TCT GTA TTG TCT T3´ (*4*) R:5´CAC CCG GTA CAA GCA GGA TT3´ (*4*)	191	6.47
**EPEC** E2348-69 O127:H6^b^ (*10*) B171-8 O111:NM (*10*) 659-79 O119:H6 (*10*) E851/71 O142:H6 (*10*)	*bfpA*	F:5´AAT GGT GCT TGC GCT TGC TGC3´ (*5*) R:5´ GCC GCT TTA TCC AAC CTG GTA3´ (*5*)	324	2.5
**EPEC** E2348-69 O127:H6^b^ (*10*) B171-8 O111:NM (*10*) 659-79 O119:H6 (*10*) E851/71 O142:H6 (*10*) **STEC** EDL933 O157:H7^b^ (*11*) TB334C O85:NM (*12*) TB285A O126:H2 (*12*) TB226A O11:HN(12)	*eaeA*	F:5´ GAC CCG GCA CAA GCA TAA GC3´ (*6*) R:5´CCA CCT GCA GCA ACA AGA GG3´ (*6*)	384	3.88
**STEC** EDL933 O157:H7^b^ (*11*) TB334C O85:NM (*12*) TB285A O126:H2 (*12*) TB226A O11:HN (*12*)	*stx1*	F:5´CTG GAT TTA ATG TCG CAT AGT G3´ ^d^ (GenBank accession no. M17358) R:5´AGA ACG CCC ACT GAG ATC ATC3´ (*6*)	150	3.88
**STEC** EDL933 O157:H7^b^ (*11*) TB226A O11:HN (*12*)	*stx2*	F:5´GGC ACT GTC TGA AAC TGC TCC3´ (*6*) R:5´TCG CCA GTT ATC TGA CAT TCT G3´ (*6*)	255	2.5
**EIEC** E11 O124NM^b^ (*13*) O124:H30 (*14*) O136:NM (*14*) O143:NM (*14*)	*ial*	F:5´GGT ATG ATG ATG ATG AGT CCA 3´ ^d^ (GenBank accession no. D13663) R:5´ GGA GGC CAA CAA TTA TTT CC 3´^d^	650	10.25

^a^*E. coli*, *Escherichia coli*; ETEC, enterotoxigenic *E. coli*; EPEC, enteropathogenic *E. coli*; EIEC, enteroinvasive *E. coli*; STEC, Shiga-toxin–producing *E. coli*; PCR, polymerase chain reaction. ^b^*E. coli* prototype strains.  ^c^Donated by the Public Health Laboratory Service, Central Health Laboratory, London, United Kingdom.  ^d^These primers were designed by us from the gene bank sequences.

We prepared bacterial lysates by resuspending single colonies in 1 mL of deionized water (Milli-Q System, Millipore, Bedford, MA), boiling them 1 min, and then freezing them until needed. *E. coli* O86:H18 was the negative control in all assays. Each PCR tube contained 23 µL of reaction mix, comprised (in final concentrations) of Tris-HCl (10 mM, pH 8.3), KCl (50 mM), MgCl_2_ (2 mM), gelatin (100 µg/mL), glycerol (5% v/v), dATP, dCTP, dGTP, and dTTP (200 µM each), Ampli*Taq* polymerase (GIBCO-BRL) (0.5 U/23 µL), a mixture of the 14 primers (Table 1), and 2 µL of bacterial lysates. The final concentration of each primer in the reaction mix was determined by employing a DNA mix (Table 1) of the four prototype *E. coli* (*7*,*10*,*11*,*13*), until each of the seven PCR products exhibited a band of similar intensity after electrophoresis in a 2.5% agarose gel in Tris-borate-EDTA buffer and ethidium staining (Figure). The solutions were then subjected to the following cycling conditions: 50°C (2 min, 1 cycle); 95°C (5 min, 1 cycle); 95°C, 50°C, and 72°C (45 sec each temperature, 40 cycles); and a final extension step (10 min, 72°C) in a thermal cycler (iCycler System, Bio-Rad Laboratories, Inc., Hercules, CA). PCR products (4 µL) were visualized after electrophoresis and ethidium staining. The PCR sensitivity was determined by suspending one colony of each reference strain in individual 1-mL aliquots of sterile saline (0.85% w/v). Serial twofold dilutions in sterile saline were then made (to 1:256), and bacterial concentrations were determined by plating on MacConkey agar. Each dilution was also subjected to PCR analysis. *E. coli* 3030 (O86:H18) strain was used as a negative control during the characterization. In all further experiments, the DNA mix from the four prototype *E. coli* served as the positive control. The multiplex PCR was further characterized by using three additional reference strains for each category (Table 1).

**Figure:**
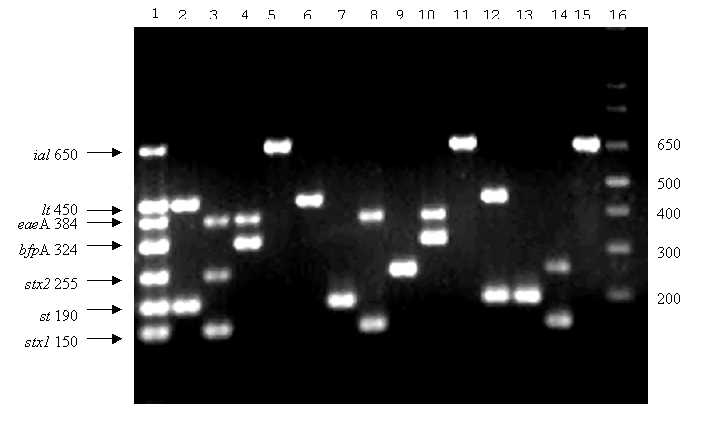
Polymerase chain reaction (PCR) products of each locus. Lane 1: sizes of the seven PCR products of each locus in base pairs, obtained when using a DNA mix of the four reference strains and the primers mix. PCR products obtained by using DNA of enterotoxigenic *Escherichia coli*, Shiga-toxin–producing *E. coli*, enteropathogenic *E. coli*, and enteroinvasive *E. coli* (lanes 2–5, respectively). Lane 6–11: PCR products obtained when using DNA of patients’ isolates and the primers mix. Lanes 12–15: PCR products obtained when using DNA of food isolates and the primers mix. Lane 16: 1 kb molecular weight marker in base pairs.

Stools from 58 children <5 years of age hospitalized for diarrhea in July, August, and September, 1999, at the three main hospitals of the Instituto Mexicano del Seguro Social, Mexico City, were studied. The Institutional Review Board of the Institute approved this study, and parental informed consent was obtained for each patient. Standard diagnostic evaluations on these stools included culture for *Campylobacter,* *Salmonella, Shigella, Vibrio cholerae, Aeromonas,* and *Plesiomonas*; identification of *Rotavirus*, *Adenoviridae*, *Astrovirus*, and *Caliciviridae* by enzyme immunoassay; and microscopy for *Entamoeba histolytica*, *Cryptosporidium parvum*, *Cyclospora cayetanensis*, *Isospora belli*, and *Giardia lamblia*. Five lactose-fermenting colonies and five sorbitol-nonfermenting colonies with morphology resembling that of *E. coli* (when present) were selected from standard and sorbitol MacConkey agar plates, respectively, speciated biochemically, and then subjected to multiplex PCR.

Because of our concern about food safety, we purchased 52 food items (hot chili sauces and taco dressings) from street vendors in Mexico City in July, August, and September, 1999, and analyzed them for the presence of *E. coli* (which indicate fecal contamination) and diarrheagenic *E. coli,* without enrichment. One gram of food was added to 1 mL of 0.85% sterile saline and vortexed, and serial 10-fold dilutions were prepared. To enumerate candidate *E. coli*, and identify diarrheagenic *E. coli*, 100 µL of each sample and dilutions were plated on MacConkey and sorbitol MacConkey agar plates. Five pink colonies from MacConkey and five colorless colonies from sorbitol MacConkey agar were tested for indole positivity and the lactose-fermenting phenotype (if selected from the sorbitol plate). Only indole-positive, lactose-fermenting colonies isolated from both media were then subjected to the multiplex PCR. STEC from patients and food were tested to determine if they expressed the O157 lipopolysaccharide antigen by using latex particle agglutination (Oxoid Limited, Basingstoke, UK Limited, Hampshire, England).

Multiplex PCR detected the appropriate loci in each positive control strain; extraneous bands were not produced (Figure). When DNA from each of the four reference strains was mixed, the same bands appeared without nonspecific amplification (Figure). The minimum number of CFU detected were 320–1,526 for ETEC; 84–168 for EPEC; 120–1,556 for EIEC; and 20–194 for *E. coli* O157:H7.

Eleven (19%) of the 58 patients had candidate diarrheagenic *E. coli* in their stools (Table 2). In 6 (55%) of these 11 patients, no other enteric pathogens was identified, and in 3 patients target sequences were found in each of the selected *E. coli* colonies (Table 2). Thus, these candidate pathogens constituted the predominant aerobic coliform flora in some samples. None of the other 47 patients with diarrhea had *E. coli* containing the target loci in their stools. Twenty-two (42%) of the 52 food samples contained *E. coli*, and 7 (13%) contained candidate diarrheagenic *E. coli* (Table 2). No STEC isolated from patients or food expressed the O157 LPS antigen, and most were *eae* negative.

**Table 2:** Diarrheagenic *Escherichia coli* isolates in patient and food samples^a^

Samples	Diarrheagenic *E. coli* group	Identified genes	No. positive strains/ no. tested	Other pathogens isolated	CFU/gram food
**Stools**					
Patient 1	STEC	*stx1,eae* A	5/5	none	
Patient 2	STEC	*stx*2	5/5	none	
Patient 3	ETEC	*lt*	2/5	none	
Patient 4	STEC	*stx* 2	2/9	none	
Patient 5	STEC	*stx* 2	1/5	none	
Patient 6	EIEC	*ial*	1/5	none	
Patient 7	ETEC	*lt*	5/5	*Shigella flexneri*	
Patient 8	ETEC	*st*	2/5	*S. sonnei*	
Patient 9	EPEC	*bfp*A*, eae*A	1/5	*S. sonnei*	
Patient 10	ETEC	*lt*	1/5	*Rotavirus*, *S. sonnei*	
Patient 11	STEC	*stx*1*, eae* A	1/10	*Rotavirus*	
**Food**					
Green sauce	ETEC	*lt, st*	5/5		8.0 X 10^2^
Green sauce	ETEC	*lt, st*	5/5		1.3 X 10^5^
Raw cabbage	STEC	*stx*1*, stx*2	2/5		2.6 X 10^5^
Green sauce	ETEC	*st*	1/5		2.6 X 10^4^
Green sauce	EIEC	*ial*	1/5		6.0 X 10^2^
Raw coriander	EIEC	*ial*	1/5		1.8 X 10^5^
Raw lettuce	EIEC	*ial*	1/5		8.2 X 10^4^

^a^STEC, Shiga-toxin–producing *Escherichia coli*; ETEC, enterotoxigenic *E. coli*; EIEC, enteroinvasive *E. coli*; EPEC, enteropathogenic *E. coli*.

## Conclusions

This multiplex PCR specifically and sensitively detected a diversity of loci in *E. coli* with ease, speed, and economy; its utility was demonstrated by using reference strains as well as clinical and food isolates. Conceivably, additional loci might be included because no signal attenuation occurred when a mixture of reference strains was assayed. The estimated cost per reaction for one strain is U.S. $2.00, compared to U.S. $15.00 for a colony blot analysis for one strain (data not shown). Furthermore, the signals from colony hybridizations are sometimes equivocal, in contrast to the unambiguous data obtained from our assay.

We believe that multiplex nucleic acid amplification to detect a panel of putatively pathogen traits should be considered as a replacement for tedious, less sensitive, and less specific detection technologies in clinical and food microbiologic analyses. This method should also be considered to be a more parsimonious use of PCR reagents than the individual locus PCR testing protocols described by others (*15*,*16*). Moreover, our approach does not rely on DNA extraction (*16*); boiling of cultures provides adequate nucleic acid to detect sequences of interest.

Comparing our protocol’s sensitivity to that reported in other protocols is difficult because of differences in methods. Specifically, other techniques seek amplicons directly from stool cultures (*17*) or employ fecal DNA extraction (*4*), whereas we assessed isolated, randomly picked colonies. Nevertheless, our sensitivity ranges were within the range of previous reports (*18*,*19*), to the extent that we were able to compare them. Our approach also provides, simultaneously, an indication of the proportion of fecal gram-negative organisms that contain loci of interest.

Without a more extensive epidemiologic analysis, we cannot state with certainty that the positive *E. coli* isolated were the causes of the diarrhea in the children studied. However, in some samples, the PCR-positive organisms were well represented among the aerobic coliform flora selected for analysis. Such organisms were also well represented among the food isolates. Because these *E. coli* indicate fecal contamination, our findings present a disconcerting picture of the hygienic status of street-vended food in Mexico City. In fact, our colony selection protocol was biased towards high-frequency organisms because we sampled only five such strains. Surveys that examine several hundred colonies (*20*) or PCR amplification of supernatant of fecal or food outgrowths (*17*,*21*) or of extracted DNA (*4*) could detect target organisms at lower densities. Though the clinical and food safety implications of low levels of candidate diarrheagenic *E. coli* remain unclear, multiple studies have demonstrated that consumption of food sold by street vendors is a risk factor for acquiring diarrhea in Mexico (*22*–*24*) and elsewhere (*25*–*27*), and attempts to improve the safety of these ubiquitous vehicles would most likely improve public health.

We have demonstrated for the first time that multiplex PCR can detect a variety of diarrheagenic *E. coli* with relative ease. Such organisms are found in food vended in Mexico City and in local children with diarrhea. This feasible technology should be evaluated in larger, controlled, prospective studies of human diarrhea and in microbiologic studies of food to establish the current epidemiology of these pathogens, including the emerging strains of STEC.
